# Quantifying intervertebral disc mechanics: a new definition of the neutral zone

**DOI:** 10.1186/1471-2474-12-38

**Published:** 2011-02-07

**Authors:** Theodoor H Smit, Manon SLM van Tunen, Albert J van der Veen, Idsart Kingma, Jaap H van Dieën

**Affiliations:** 1 VU University Medical Center, Department of Orthopaedic Surgery, Research Institute MOVE, Amsterdam, The Netherlands; 2 VU University Medical Center, Department of Physics and Medical Technology, Research Institute MOVE, Amsterdam, The Netherlands; 3 Research Institute MOVE, Faculty of Human Movement Sciences, VU University Amsterdam, The Netherlands

## Abstract

**Background:**

The neutral zone (NZ) is the range over which a spinal motion segment (SMS) moves with minimal resistance. Clear as this may seem, the various methods to quantify NZ described in the literature depend on rather arbitrary criteria. Here we present a stricter, more objective definition.

**Methods:**

To mathematically represent load-deflection of a SMS, the asymmetric curve was fitted by a summed sigmoid function. The first derivative of this curve represents the SMS compliance and the region with the highest compliance (minimal stiffness) is the NZ. To determine the boundaries of this region, the inflection points of compliance can be used as unique points. These are defined by the maximum and the minimum in the second derivative of the fitted curve, respectively. The merits of the model were investigated experimentally: eight porcine lumbar SMS's were bent in flexion-extension, before and after seven hours of axial compression.

**Results:**

The summed sigmoid function provided an excellent fit to the measured data (r^2 ^> 0.976). The NZ by the new definition was on average 2.4 (range 0.82-7.4) times the NZ as determined by the more commonly used angulation difference at zero loading. Interestingly, NZ consistently and significantly decreased after seven hours of axial compression when determined by the new definition. On the other hand, NZ increased when defined as angulation difference, probably reflecting the increase of hysteresis. The methods thus address different aspects of the load-deflection curve.

**Conclusions:**

A strict mathematical definition of the NZ is proposed, based on the compliance of the SMS. This operational definition is objective, conceptually correct, and does not depend on arbitrarily chosen criteria.

## Background

**Figure 1:**
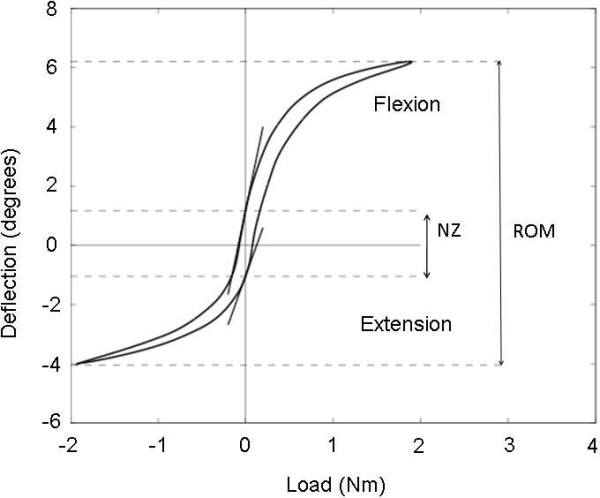
**A typical load-deflection curve from experimental data of a flexion-extension experiment on a goat lumbar spine segment**. The range of motion (ROM) is the total range of deformation upon maximal loading (+/- 2 Nm in this example). The neutral zone is the area with the least internal resistance against bending, thus the steepest slope of the load-displacement curve. NZ indicates the neutral zone as defined by Wilke et al[7]. In this concept, the neutral zone stiffness is the inverse tangent to the curve at the intersection points with the y-axis (straight lines).

The motion segment is the smallest functional unit of the spine[1]. The spinal motion segment (SMS) is deformed under the various loads of daily activities, typically described as flexion-extension, torsion, lateral bending, shear, and compression. The mechanical properties of spinal motion segments can be derived from load-deflection curves (Figure 1). One example of such a property is the range of motion (ROM), which is defined as the deflection difference between the maximum applied loads in each direction (Figure 1). Another quantification used in the load-deflection curve is the neutral zone (NZ), described as the range over which a motion segment moves with minimal resistance [1,2]. This range increases with (mild) degeneration of the intervertebral disc[3]. Excessive intervertebral motion may result in pain due to overstretching of soft tissues and/or entrapment of nerve roots. Clinically, excessive motion is referred to as spinal instability, which may result from, among others, degenerative disc disease and spondylolisthesis. Degeneration of the intervertebral disc is routinely assessed radiologically, but it appears to be poorly related to pain and instability[4]. The NZ offers a more direct measure of spinal instability and more recently techniques have been developed to estimate this parameter in vivo[5]. The NZ was found to be more sensitive than the ROM in quantifying spinal destabilization (*e.g*. caused by spondylolisthesis[5,6]).

Panjabi [2] operationally defined the NZ based on the finding that a motion segment does not return to its initial position after loading in a particular direction. This residual displacement, which was measured 30 seconds after removal of the load, was equated with the magnitude of the NZ. The NZ is thus seen as play in the SMS when moving from one direction to the other, which is essentially different from the original definition as the zone of minimal stiffness[2]. Residual displacement results from the visco- and poro-elastic (*i.e*. time-dependent) properties of the intervertebral disc, which cause the SMS to creep under load. This implies that the magnitude of the NZ, given this operational definition, depends on the measurement time after removal of the load as well as on the loading history before relaxation. Both the maximum load applied, and thus the loading history, and the point in time at which the residual displacement is measured, are arbitrarily chosen.

Where Panjabi used static residual displacements for determining the magnitude of the NZ, Wilke and colleagues determined the NZ in dynamic loading experiments[7]. The NZ was defined as the angulation difference at zero load between the two directions of motion (Figure 1). The continuous motion also allows defining the neutral zone compliance, which was defined as the tangent to the curve at zero load in both directions. The advantage of this method is that the NZ is derived from the load-deflection curves without involving any arbitrary choices, but the deformation at zero load does not per se mark the borders of minimal segment stiffness. For example, the spinal motion segment may not have been embedded accurately in its neutral position, thereby shifting the zero-load condition towards one end of the ROM, resulting in a different-presumably smaller- NZ region. In the case of sagittal bending where asymmetry exists in flexion and extension, one may in fact question where the neutral position of a SMS actually is. More importantly, it is not clear what the angulation difference at zero load physically means: it is tempting to call it the backlash of the SMS, but that would imply that the SMS can move freely between the given boundaries of the NZ. This is not the case, because the NZ stiffness is not zero. Instead, like Panjabi's operational definition for the NZ, the angulation difference is a consequence of the hysteresis of the SMS, which in turn depends on the loading history of the poro-visco-elastic structure.

Spenciner and colleages [8] defined the borders of the neutral zone as the intersection of the y-axis (zero load) and the tangent line to the load-deflection curve drawn at the point of movement reversal (maximum load). With this method, the NZ obviously depends on the maximum load applied and thus the ROM. When the applied maximum load is smaller, the slope of the curve will be larger, causing a steeper slope of the tangent line, which results in a smaller NZ. Thus, the stiffness at the reversal points is taken into account but not the stiffness of the NZ.

Sarver and Elliott[9] defined the neutral zone for tension-compression loading using a tri-linear fit to the load-deflection curve. The middle region of the best fit, with the steepest slope (high compliance) was defined as the neutral zoned. However, the kind of data obtained under bending and torsion loading cannot often be fitted well with a tri-linear function, as clear transition zones are present in load-deflection curves under bending. (Figure 1).

Thompson and colleagues[10] explicitly defined the NZ in terms of minimal stiffness. They fitted a fourth degree polynomial over the load-deflection data. The stiffness was then simply obtained as the first derivative of this polynomial and the borders of the NZ were defined at 0.05 Nm/° and -0.05 Nm/°. There are two points of concern with this approach. First, arbitrary boundaries for the minimal stiffness region are used. Secondly, it appears that the borders are defined in such a way that a NZ cannot always be defined because the first derivative does not always exceed the thresholds of 0.05 Nm/° and -0.05 Nm/° (data not shown).

It is generally accepted that the NZ of a spinal segment is defined as the region of minimal stiffness. However, only the methods by Sarver and Elliot and Thompson et al. correctly consider the SMS stiffness (or its inverse: the compliance) to determine the boundaries of the NZ region. We built on these methods by developing an operational definition of the NZ based on a mathematical model of the load-deflection data, that is: free from the limitations mentioned above. Thus, the general aim of the paper is to present and evaluate a strict mathematical definition of the Neutral Zone of a spinal motion segment. In order to evaluate the new method for determining NZ magnitude and stiffness, we performed experimental biomechanical testing on porcine lumbar motion segments. The specific aims of these experiments were [1] to determine the degree of fit of a newly defined function to the load-deflection data of SMS and the degree of linear fit over the load-deflection data within the NZ to estimate NZ stiffness; [2] to establish the robustness of the method for the direction of loading (flexion-extension vs. extension-flexion); [3] to compare calculated NZ and NZ stiffness to the angulation difference at zero load and the related stiffness as defined by Wilke; [4] to explore whether NZ magnitude as defined by the angulation difference at zero load indeed reflects hysteresis; and [5] to determine the effect of compressive loading on NZ, NZ stiffness, and hysteresis. The general hypothesis is that the two methods will provide different results, since they represent different underlying concepts. In addition, we hypothesized that NZ magnitude as defined by the angulation difference at zero load is correlated to hysteresis, because this actually measures the distance between the flexion-extension and extension-flexion curves at zero load. With respect to the effect of compressive loading, we hypothesized in line with literature[11] that a period of axial compression decreases the NZ magnitude of the SMS as estimated with our method, while it increases the hysteresis. Given the assumed relationship of the angulation difference at zero load with hysteresis, we also hypothesized the NZ as determined with Wilke's method to be increased after axial loading.

## Methods

### Mathematical model

Practically, there is generally too much noise in the raw load-deflection data to numerically determine compliance, which is the first derivative of the load-deflection curve. Also the fact that data points are not necessarily equally spaced in time makes data processing harder. To overcome these problems, we propose to mathematically fit the experimental load-deflection data. The mathematical fit filters the noise and allows for an analytical calculation of the non-linear segment compliance. To create a mathematical model, attention has to be paid to several points. First, we look for the region of minimal stiffness. This implies that the borders of the NZ must be objectively defined in terms of stiffness or its inverse, compliance. Thus, the NZ should not be dependent on the arbitrarily chosen maximum load. Secondly, the asymmetry of the load-deflection curve should be taken into account, because of the asymmetrical structure of the intervertebral disc. Finally, the NZ should be independent from the starting point of the load-deflection curve or the position of the spinal segment after embedding. As a full load-deflection curve shows hysteresis due to poro-visco-elastic behaviour (Figure 1), the fit should be made once over the curve along one loading direction (e.g. full flexion to full extension) and once over the curve along the other loading direction (e.g. full extension to full flexion).. The analysis thus results in two values for both, the neutral zone magnitude and its stiffness, which are hypothesised to be equal.

**Figure 2:**
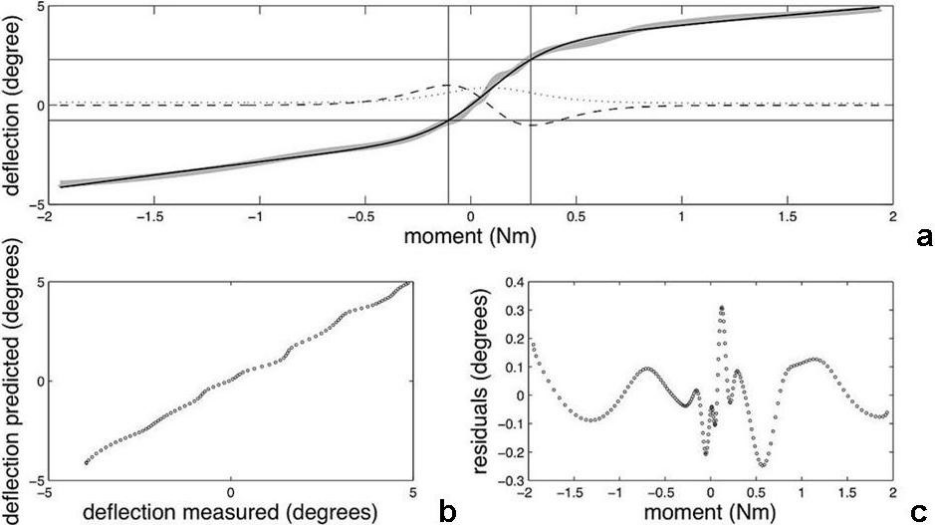
**The fitted double sigmoid function**. a. Load-deflection data (gray) are fitted with a double sigmoid function (black). The first derivative indicating the compliance is shown as a dotted line. At the maximum and minimum of the second derivative (dashed line) vertical lines are drawn delineating NZ. Goodness of fit (b.) and residuals plot (c.) show the quality of the double sigmoid model for describing the mechanical behaviour of the spinal motion segment. The data in both lower panels were down-sampled for clarity.

There exists no theory that prescribes the type of function, which describes the mechanical behaviour of the segment (*e.g*. under flexion-extension). However, it is important to note that the NZ exists and actually only can be defined by virtue of the characteristic S-shape of the load-deflection curve. The double sigmoid function has such a shape and was found to provide an excellent fit over the entire curve (Figure 2):

(1) $$D=\frac{1}{1+e^{-(a1+b1L)}}*c1+\frac{1}{1+e^{-(a2+b2L)}}*c2+d$$

Deflection D of the motion segment is expressed as a function of external load L. The sigmoid function is chosen because of its S-like shape and it is summed to allow for the natural asymmetry in the segment's flexion-extension curve. The parameters a and d in the equation determine the location of the load-deflection curve by shifting it horizontally (along the x- or loading axis) and vertically (along the y- or deflection axis), respectively. Parameters b and c reflect the biomechanical properties of the spinal motion segment. Parameter b determines the slope of the curve and thus the NZ stiffness. Parameter c determines the range of motion (ROM) of the segment but by changing the slope of the curve also the NZ stiffness. Eq.1 was fitted to experimental data, using unconstrained nonlinear minimization of the root mean squared error in a custom made matlab (the Mathworks Natick MA, USA) routine, employing the "fminsearch" function from the matlab optimization toolbox.

The NZ is that part of the load-deflection curve where compliance is maximal. The summed sigmoid function (Eq.1) can be used to determine compliance analytically as the first derivative (dD/dL; Figure 2). To obtain the range of maximal compliance (or minimal stiffness, *i.e*. the NZ), the *change *in compliance should be determined, which can be done with the second derivative (d^2^D/dL^2^). This curve provides unique inflection points that indicate the beginning and the end of the NZ, defined by the maximum and minimum in the second derivative (Figure 2).

### Neutral Zone Stiffness

The neutral zone stiffness itself is defined by the inverse of the compliance, which in turn is defined by the slope of the load-deflection curve over the full range of the NZ. To quantify this, a straight line was fitted over the load-deflection curve in the range of the NZ.

### Experimental data

**Figure 3:**
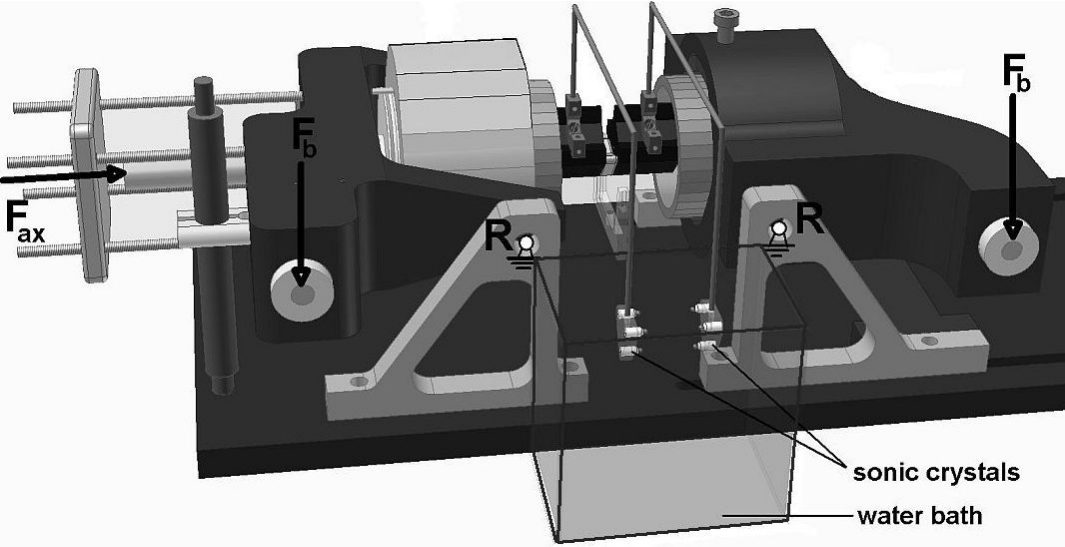
**Sketch of the experimental set-up for four-point bending**. The spinal motion segment (black) is embedded in the segment holder. Each vertebral body is provided with a jib holding three sonic crystals each. For communication, the crystals are submerged in a water bath. Bending forces F_b _are applied by a materials testing device (not shown) and the segment holders rotate around rotation points R. Axial compression can be applied by a hydraulic force F_ax_.

To test the new mathematical definition of the NZ, we used experimental load-deflection curves from other (unpublished) studies. These load-deflection curves were determined from eight porcine lumbar spinal motion segments, which were loaded in flexion and extension to a maximum of 2 Nm in each direction. To this end, segments were embedded in woods metal and mounted on a four-point bending device controlled by a hydraulic material testing machine (Instron 8872, High Wycombe, UK)[12,13] (Figure 3). Bending moments were applied by a constant rate displacement of the cross-head of the materials testing machine, providing a bending rate of 1.5°/s. During the experiments, the segments were kept wet by frequently spraying saline. The position of the vertebrae was determined with a Sonometrics Digital Ultrasonic Measurement System (Sonometrics Corporation, London, Canada), consisting of piezo-electric crystals sending and receiving pulses. Three crystals were mounted on a jib attached to each vertebra and submerged in a water bath (Figure 3). Three more crystals were connected to earth for reference. The distance between the crystals was calculated from the time difference between sending and receiving the pulses. Custom-made software was used to calculate vertebral motions from the distance between the separate crystals. In line with the recommendations by Wilke et al[7]., the third loading cycle in each experiments was used to quantify the NZ magnitude and stiffness.

### Compressive loading

To determine the effect of prolonged axial loading, 250N of axial compression was applied on the same eight segments for a period of seven hours (Figure 3). During this period, the specimens were wrapped in a saline soaked cloth to prevent dissication. After removal of the axial load the experiments were repeated. NZ magnitude and stiffness were calculated by both, the double sigmoid function and the angulation difference. Hystersis of the segment was calculated from the area between the flexion-extension and extension-flexion curves.

### Statistics

Goodness of fit of the double sigmoid function to experimental load-deflection data of procine SMS tested before and after 7 hours of axial loading was quantified by means of the coefficient of determination (r^2^, the square of Pearson's coefficient of correlation) between predicted and measured defection data. Linearity of the load-deflection data in the NZ was evaluated by the coefficient of determination (r^2^) of the linear least-squares fit.

To test to what extent the same results were obtained for NZ magnitude and NZ stiffness from flexion-extension and extension-flexion curves, the data were compared by means of paired t-tests and in addition the intra-class correlation (ICC) between these observations was determined. An ICC < 0.60 was qualified as poor, 0.60≤ ICC < 0.80 as moderate and ICC≥0.80 as good.

Next, the results were averaged for both movement directions and the NZ magnitudes and NZ stiffness obtained with the proposed method were compared to those obtained with the method of Wilke[7] using paired t-tests. In addition, the extent to which the two methods yielded common information was expressed by means of Pearson's coefficient of correlation.

The effect of axial loading on NZ magnitude and NZ stiffness as determined with both methods was determined using paired t-tests and a similar comparison was made between the values for hysteresis before and after loading.

## Results

### Goodness of fit

In all experiments, the summed sigmoid function provided an excellent fit to the measured load-deflection curve with r^2 ^> 0.976 (Figure 2). The NZ appeared to be characterized by a nearly constant stiffness, as a linear fit yielded r^2 ^> 0.997 for all tests.

### NZ ad NZ stiffness determined with the proposed method

NZ magnitudes obtained in extension-flexion and flexion-extension averaged 2.57 (SD 1.05) and 2.52 (SD 0.90) degrees, respectively and were not systematically different (p = 0.766). In addition, the ICC between these values was 0.739 indicating moderate correspondence. The NZ magnitude according to the double sigmoid function was not correlated with hysteresis (r = -0.194; p = 0.472),

The NZ stiffness determined in extension-flexion and flexion-extension averaged 0.138 (SD 0.060) and 0.133 (SD 0.067) Nm/degree, respectively, without a systematic difference (p = 0.394) and an ICC of 0.971, indicating good correspondence. For further analysis values were averaged over both movement directions. The correlation between NZ magnitude and NZ stiffness of the new method was low and not significant (r = -0.489, p = 0.055).

### Angulation difference at zero load

On average the NZ magnitude operationally defined by Wilke as the angulation difference was 1.49 (SD 0.93) degrees. As hypothesized, the NZ magnitude was correlated to the hysteresis (r = 0.726, p = 0.001). This method yields two estimates of NZ stiffness, which averaged 0.125 (SD 0.029) and 0.103 (SD 0.027) Nm/degree in extension-flexion and flexion-extension, respectively. The difference was small but significant (p = 0.026) and the two estimates were poorly correlated with an ICC of 0.203. Nevertheless, for further analysis stiffness estimates were averaged over both movement directions.

### Comparison between methods

**Figure 4:**
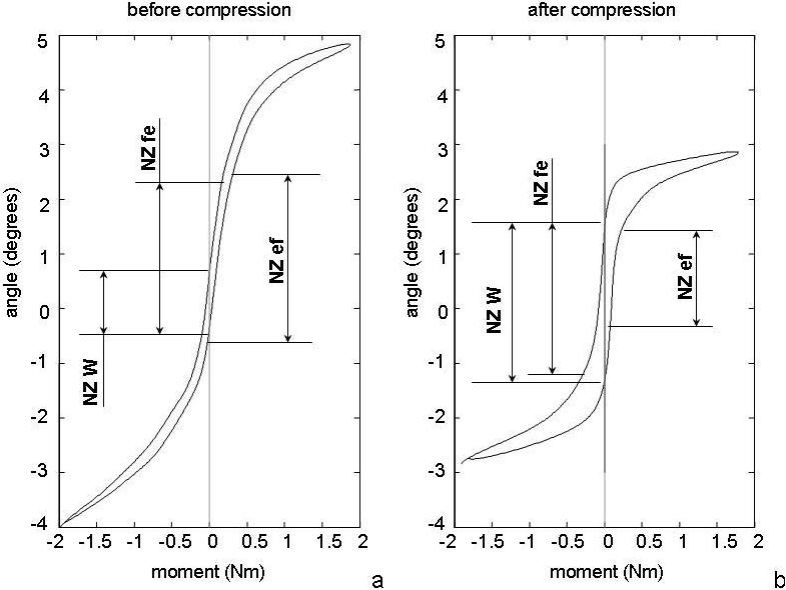
**Determination of the NZ from experimental data**. Load-deflection curve of a porcine lumbar segment before (a) and after a seven-hour period of axial compression under 250N (b). The NZ as determined by the method of Wilke (NZW) underestimates the NZ before the compression test, because the range of minimal stiffness is clearly larger than the angulation difference at zero load (a). The NZ as determined by the double sigmoid function (NZef, NZfe) appear to present a better estimation. After a seven-hour axial compression, the range of motion (ROM) of the segment has decreased and hysteresis has increased (b). The angulation difference at zero load (NZW) is larger than the NZ determined by the double sigmoid function. Note that in this case the NZ for extension to flexion (NZef) is smaller than the NZ from flexion to extension (NZfe).

**Figure 5:**
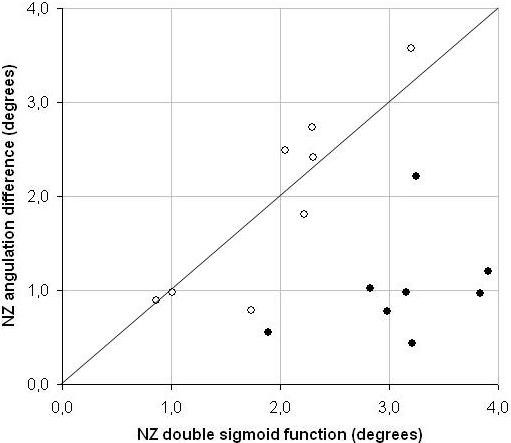
**Scatter plot of the Neutral Zone magnitudes**. The NZ calculated by the double sigmoid function (x-axis) as compared to the angulation difference as defined by Wilke (y-axis). Before the conditioning by continuous axial compression, the NZ as determined with the double sigmoid function is considerably larger than the angulation difference (closed circles). After a seven hours compression period, the values are more comparable (open circles). The thin line represents identity.

Estimates of NZ magnitude obtained from the summed sigmoid function were significantly larger than the average NZ magnitude defined by angulation difference at zero load (F_1,7 _= 37.5, p < 0.001; Figure 4a and 5). In addition, the NZ magnitudes according to both methods were uncorrelated (r = 0.090; p = 0.355). The NZ stiffness was slightly but not significantly smaller when determined through the double sigmoid function (0.136 (0.062) vs. 0.154 (0.097) Nm/degree; p = 0.380). The values were in addition only poorly and not significantly correlated (r = 0.487; p = 0.056; Figure 5).

### The effect of compressive loading

The load-deflection curves of all segments had changed considerably after axial compression at 250 N for seven hours (Figure 4). The ROM consistently decreased from an average of 8.23 (SD 1.28) to 4.73 (SD 1.07) degrees (p < 0.00001). Despite the lower ROM, the hysteresis of the spinal motion segments increased from 0.93J before axial compression to 1.24J after axial compression (+33%, p = 0.0215). Interestingly, the NZ magnitude as determined from the double sigmoid function consistently and significantly decreased (-1.17 ± 0.73°, p < 0,0001), but it consistently and significantly increased when determined by the angulation difference (+0.94 ± 0.86°, p = 0.0178). Furthermore, and in contrast with the situation before compressive loading, the NZ as determined by both methods were quite comparable after seven hours of compression (both 1.96°, p = 0,98) and they were highly correlated (r = 0.893; p = 0.003; Figure 5).

**Figure 6:**
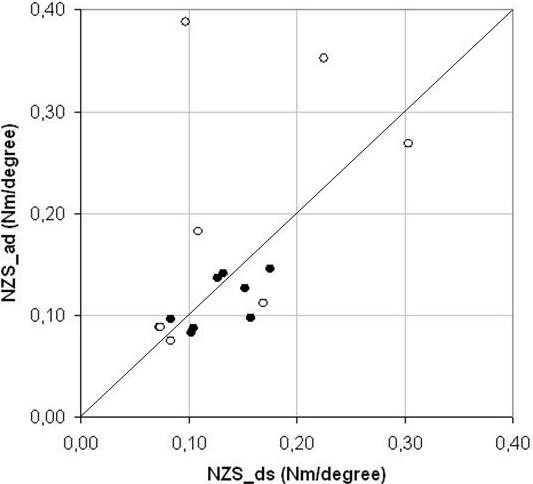
**Scatter plot of the Neutral Zone stiffnesses**. The NZ stiffness calculated by the double sigmoid function (NZS_ds, x-axis) as compared to the angulation difference as defined by Wilke (NZS_ad, y-axis). The stiffness appears to be more consistent before preloading (closed circles) as compared to after preloading (open circles). Note the three outliers from the loaded segments. The thin line represents identity.

Finally, the NZ stiffness averaged over both loading directions was not significantly different before and after axial compression, for either method (0.129 ± 0.032 Nm/° vs. 0.142 ± 0.084 Nm/° (p = 0.64) for the double sigmoid function; 0.114 ± 0.026 Nm/° vs. 0.194 ± 0.126 Nm/°, (p = 0.084) when determined by the angulation difference; Figure 6).

## Discussion

In this study, we propose a new operational definition of the neutral zone (NZ), the region of minimal stiffness or maximal compliance of a spinal motion segment, with an objective, mathematical method. Building on existing methods, this definition is based on the mechanical properties of the spinal motion segment and it does not depend on arbitrary choices in the analysis or on orientation of the segment after embedding. A summed sigmoid is fitted over the data and the maximum and minimum of the second derivative are used as the unique and objective borders of the NZ. The summed sigmoid function provided an excellent fit to experimental load-deflection data of porcine SMS. The neutral zone defined according to the new method was characterized by a nearly linear load-deflection relationship. When applied to freshly thawed lumbar spines, the magnitude of the neutral zone according to the new mathematical definition was significantly larger than and not correlated to the angulation difference at zero load, the most commonly used operational definition of the NZ proposed by Wilke[14-18]. Our measurements showed that the NZ magnitude is strongly influenced by loading history: a 7-hour continued axial compression decreased the NZ magnitude as determined with the new method, but increased the angulation difference at zero load as determined by Wilke's method, resulting in a remarkably similar NZ magnitude for both methods. The NZ stiffness was not dependent on method, nor on loading history.

It should be emphasised that no theory exists which prescribes the type of curve to be used for fitting the load-deflection data. However, it also should be noted that the NZ exists by virtue of the S-shaped load-deflection curve typically observed in bending- and torsion experiments. Several other mathematical functions (*e.g*.: polynomials, exponential functions) could (and in fact have been[10]) tested as well, but the summation of two sigmoids captures both the S-shape and the asymmetry of the typical SMS load-deflection curve and provides coefficients that are interpretable in mechanical terms. Despite the excellent fit of the summed sigmoid function (r^2 ^> 0.976), it should be emphasized that it is still no more than an approximation of the real load-deflection curve. Its advantages are that it disregards the noise of the experimental data and it allows for an analytical calculation of the zone with the least stiffness. Other curve types may do that as well or even better, crucial is that by curve fitting, the NZ and its stiffness can objectively be derived from the experimental data in a way that is consistent with the definition of the neutral zone and with the typical asymmetrical S-form of the load-deflection curve.

**Figure 7:**
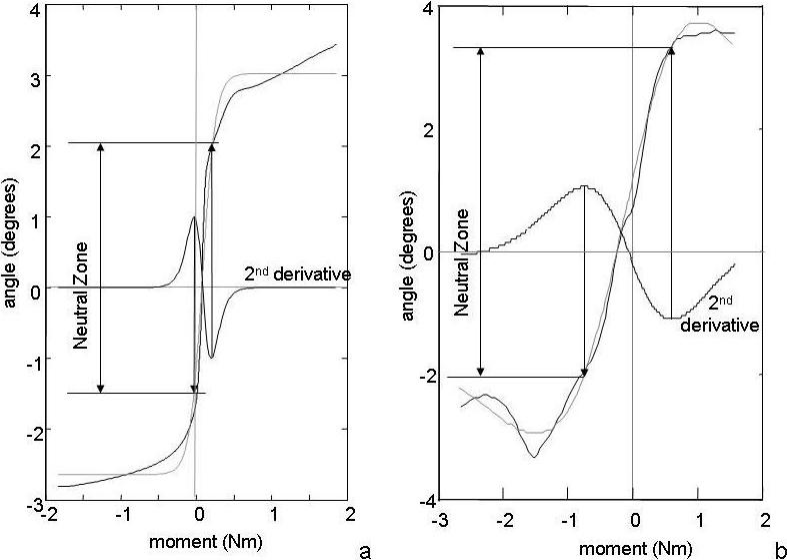
**Data fitting in suboptimal load-deflection curves**. a. the worst fit in the validation experiments of this study with porcine lumbar motion segments (r^2 ^= 0.976). The double sigmoid function (gray) is not able to follow the entire experimental load-deflection curve (black). Yet the fitted function allows assessing mathematically the deflection point of the curve and thereby the NZ. b. Load-deflection curve recorded from a human specimen from another study[12], showing a non-monotonous curve at 1.5 Nm extension load (probably due to local failure) and a non-linear range of the NZ. The double-sigmoid function (gray) fits reasonably well over the NZ and allows objective determination of its borders by the maximum and minimum of the second derivative.

The summed sigmoid function approached the experimental data quite well, but not always for the full range of motion (Figure 7a). This means that Eq.1 does not fully cover the SMS load-deflection behaviour, especially at higher bending loads. It is unclear, however, what this practically means for the calculation of the NZ magnitude and stiffness: even for the worst fit in our series, NZ magnitude and stiffness seem reasonably well estimated (Figure 7a). Apparently, the double sigmoid function correctly describes the inflection points of compliance, which define both magnitude and stiffness of the NZ. Higher order functions like a triple sigmoid would probably improve the goodness of fit, but also increases the risk of fitting artefacts.

The application of our model to data drawn from experiments with young pigs may mask a limitation of the method: experienced experimenters know that older and more pathological (human) specimens may exhibit a much more discontinuous response (Figure 7b), as would specimens that have been experimentally destabilized, with clear and abrupt inflection points in the loading and unloading curves. Such data, however, may be difficult to interpret. Discontinuities may arise from measurement artefacts like suboptimal embedding or slip-stick within the testing device. While such errors should be avoided, they may be difficult to recognize even for an experienced experimenter. Furthermore, discontinuities may occur as a result of impingement of the vertebrae, or due to tissue damage in the specimen. It appears that curves still may fit very well, but the interpretation becomes more questionable: for example, what does a local maximum within the neutral zone really mean? The newly proposed method nor any other method can answer such questions. The neutral zone as also defined by others assumes a more or less smooth, asymmetrical S-shaped load-deflection curve. As such, the new method presents an excellent way of quantifying magnitude and stiffness of the neutral zone. Lack of fit can easily be flagged and if data cannot be fitted properly, results should be interpreted with care.

The load-deflection curves before and after a seven-hour period of axial compression showed an interesting difference between the NZ as defined by the double sigmoid function and the angular deflection at zero load as defined by Wilke et al.[7] NZ magnitude decreased when based on stiffness considerations, but increased when based on angular difference at zero loads. Both parameters thus represent another aspect of SMS behavior. As can be appreciated from Figure 4, the distance between the flexion-extension curve and the extension-flexion curve has increased. Accordingly, we found a consistent increase in hysteresis, despite the strong decrease in the range of motion. The angular deflection at zero load thus appears as a measure of hysteresis of the load-deflection curve and in fact a moderate positive correlation was found. Why the hysteresis increases while the NZ magnitude decreases, is not quite understood, but it is in line with earlier reports[11]. Presumably, axial compression released interstitial fluid from the specimens, thereby leaving the intervertebral disc more viscous than before axial compression was applied.

Interestingly, the NZ magnitude after the seven hour compression period was remarkably similar for both methods: the magnitude was the same (1.96° p = 0.98) and they were highly correlated (r = 0.893; p = 0.003; Figure 5). This would indicate that the methods are interchangeable, but the other findings in the current study show that this is not the case: the angulation difference is both conceptually and numerically different from the stiffness derived NZ magnitude. In contrast to other reports, we only found an insignificant increase in NZ stiffness after axial compression when determined by Wilke's method, and no increase when determined by the double sigmoid function. We suggest that this occurred because we released compression just before testing; which resulted in a situation in which the disc contained less fluid, but the fibers of the disc were released as in the original situation before axial compression had been applied.

All methods reviewed in the literature define only one NZ, whereas we defined a NZ for both directions of the load-deflection curve. This is inherent to the fact that poro-visco-elastic behaviour of the spinal segment is reflected in a hysteresis loop. However, NZ magnitudes in flexion and extension appeared to be similar and quite well correlated (see Figure 5a for an illustration of this and Figure 5b for a counterexample). Practically, one thus might define the NZ magnitude as the NZ magnitude in either direction, or alternatively as their average. The same applies to the NZ stiffness. We urge future users to report both NZ magnitude and NZ stiffness, as these are more or less independent values (r^2 ^= 0.24) and provide valuable information on the mechanical behaviour of the spinal motion segment.

## Conclusions

A strict mathematical definition was proposed to objectively quantify the size of the neutral zone as well as its stiffness. In contrast to existing methods, this definition is based solely on the mechanical properties of the spinal motion segment and it does not depend on arbitrary choices in thresholds or orientation of the segment after embedding. Furthermore, we showed that this method is sensitive to changes in the loading history of the segment. We hope this method will prove useful to more correctly quantify spinal segment behaviour.

## Competing interests

The authors declare that they have no competing interests.

## Authors' contributions

TS conceived the new definition of the neutral zone and drafted the manuscript; MT co-performed the experiments and wrote the software for automatically determining the neutral zone; AJV performed the experiments; IK performed the data processing and finalized the software developed; JHD conceived the method, supervised the software development and drafted the manuscript. All authors read and approved with the manuscript.

## Pre-publication history

The pre-publication history for this paper can be accessed here:

http://www.biomedcentral.com/1471-2474/12/38/prepub
